# Collaborative Depression Trial (CADET): multi-centre randomised controlled trial of collaborative care for depression - study protocol

**DOI:** 10.1186/1472-6963-9-188

**Published:** 2009-10-16

**Authors:** David A Richards, Adwoa Hughes-Morley, Rachel A Hayes, Ricardo Araya, Michael Barkham, John M Bland, Peter Bower, John Cape, Carolyn A Chew-Graham, Linda Gask, Simon Gilbody, Colin Green, David Kessler, Glyn Lewis, Karina Lovell, Chris Manning, Stephen Pilling

**Affiliations:** 1 Mood Disorders Centre, School of Psychology, University of Exeter, EX4 4QG, UK; 2 Academic Unit of Psychiatry, University of Bristol, Cotham Hill, BS6 6JL, UK; 3 Clinical Psychology Unit, Dept of Psychology, University of Sheffield, S10 2TP, UK; 4 Department of Health Sciences, 1st floor, Seebohm Rowntree Building, University of York, Heslington, York, YO10 5DD, UK; 5 NPCRDC, Williamson Building, University of Manchester, Oxford Road, Manchester M13 9PL, UK; 6 Camden and Islington Mental Health and Social Care Trust, East Wing, St Pancras Hospital, 4 St Pancras Way, London, NW1 0PE, UK; 7 University of Manchester, School of Community Based Medicine, Rusholme Academic Unit, Walmer Street, Manchester, M14 5NP, UK; 8 Peninsula Medical School, University of Exeter, St Lukes Campus, Exeter EX1 2LU, UK; 9 Academic Unit of Primary Health Care, University of Bristol, 25 Belgrave Road, Clifton, Bristol BS8 2AA, UK; 10 The School of Nursing, Midwifery and Social Work, University of Manchester, Room 6.322a, Jean McFarlane Building, University Place, Oxford Road, Manchester, M13 9PL, UK; 11 Upstream Healthcare Ltd, Unit 5, 2a, Laurel Avenue, Twickenham, TW1 4JA, UK; 12 CORE, Clinical Health Psychology, 1-19 Torrington Place, London, WC1E 7HB, UK

## Abstract

**Background:**

Comprising of both organisational and patient level components, collaborative care is a potentially powerful intervention for improving depression treatment in UK primary Care. However, as previous models have been developed and evaluated in the United States, it is necessary to establish the effect of collaborative care in the UK in order to determine whether this innovative treatment model can replicate benefits for patients outside the US. This Phase III trial was preceded by a Phase II patient level RCT, following the MRC Complex Intervention Framework.

**Methods/Design:**

A multi-centre controlled trial with cluster-randomised allocation of GP practices. GP practices will be randomised to usual care control or to "collaborative care" - a combination of case manager coordinated support and brief psychological treatment, enhanced specialist and GP communication. The primary outcome will be symptoms of depression as assessed by the PHQ-9.

**Discussion:**

If collaborative care is demonstrated to be effective we will have evidence to enable the NHS to substantially improve the organisation of depressed patients in primary care, and to assist primary care providers to deliver a model of enhanced depression care which is both effective and acceptable to patients.

**Trial Registration Number:**

ISRCTN32829227

## Background

Depression is a major health problem causing substantial disability and set to become the second largest cause of global disability by 2020 [1,2]. Despite the availability of effective pharmacological and psychological treatments for depression, patients often receive a less than optimal treatment programme. In primary healthcare systems internationally, patient adherence with pharmacological treatment is poor [3] and problems are exacerbated further by organisational barriers between generalist and specialist mental health professionals [4,5]. Generalist primary care physicians often have very limited support when helping patients with both pharmacological treatment and psychosocial interventions. Such support may be critical given that in systems such as that in the United Kingdom (UK) and elsewhere, the general practitioner (GP) is the sole responsible medical clinician for 90-95% of patients [6].

Attempts to improve this situation have seen the development of organisational strategies including increased resources to specialist services, education of primary care clinicians, consultation liaison services and stepped care [7]. A systematic review of 36 organisational intervention studies concluded that simple models such as guidelines and education were ineffective in improving the management of depression [8]. Gunn et al [9] identified the components of effective organisational quality improvement strategies as: a multi-professional approach to patient care; a structured management plan; scheduled patient follow-ups; and enhanced inter-professional communication.

One model which has seen an increasing efficacy and effectiveness literature is 'collaborative care' [7] which highlights the chronic nature of depression and proposes that the whole system of care for depression needs to be reengineered. Collaborative care is a complex combination of clinician and patient education, consultation-liaison between primary and secondary care clinicians and case management [10], translated into practice by the introduction of a new case manager role into primary care who liaises between primary care clinicians and mental health specialists, collects and shares information on the clinical care of individual patients and delivers and manages aspects of their care [7].

Whilst collaborative care improves outcomes over usual care [11-13], the vast majority of models have been developed and evaluated in the United States (US) [14]. Given this, it is necessary to establish the international generalisability of collaborative care to determine if these outcomes can be replicated beyond the US, where the nature of patient populations and patterns of service utilization may differ. The feasibility and acceptability of implementation in the UK National Health Service (NHS) is likely to be shaped by funding arrangements, deployment of staff and the structure and organization of component parts of the NHS (particularly primary care).

In order to investigate collaborative care in the UK, we adopted the UK Medical Research Council's (MRC) [15,16] strategy for the investigation of complex interventions. Through a series of exploratory qualitative and quantitative studies as part of a 'Trial Platform' funded by the Medical Research Council [17-20] we found collaborative care to have a moderate to large effect (0.63; 95% confidence interval = 0.18 to 1.07), the first time this has been demonstrated in the UK, with change in PHQ-9 scores achieved by the intervention patients from baseline to follow up equating to a clinical shift of almost two categories of depression severity. We also found that the best method of recruitment was through case finding and that collaborative care was acceptable to patients and mental health workers. As a consequence, we have designed a fully powered trial of collaborative care as the next step in our phased approach to investigating this complex intervention.

## Methods/Design

### Objectives

Is collaborative care more clinically and cost effective than usual care in the management of patients with moderate to severe depression in UK primary care?

### Study Design

**Figure 1:**
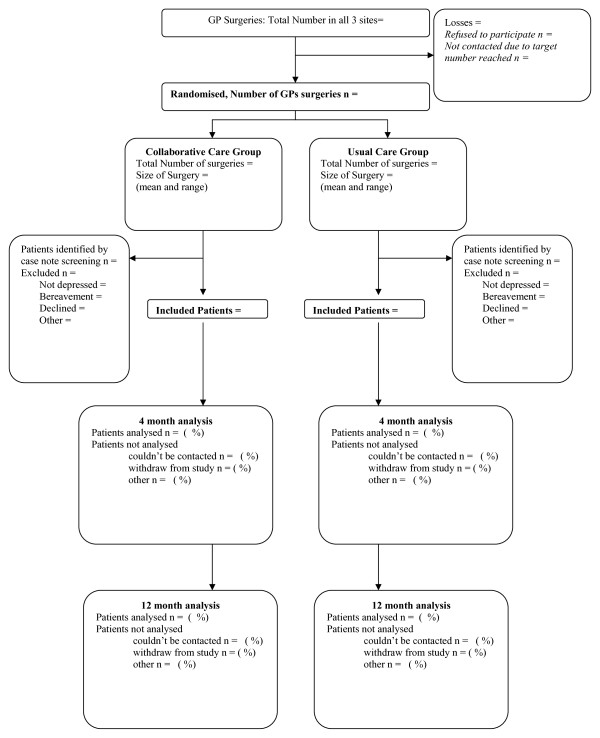
**Consort diagram detailing progression through the trial**.

This is a pragmatic cluster randomised controlled trial with allocation of clusters (GP practices) to two alternative branches (see Figure 1)

1) Collaborative care (experimental group)

2) Usual management of depression (control group)

The rationale for a cluster randomised trial comes from data collected in our trial platform [19] where we tested directly for the presence of contamination between experimental and control conditions. In this pilot phase, after initially randomising General Practices to treatment or cluster control conditions, patients in the treatment cluster group were then individually randomised to either collaborative care or usual care control. This created three study groups (cluster-randomized controls, individually-randomized intervention patients, individually-randomized control patients). Our results showed evidence for substantial contamination, in that when compared to the intervention group's outcomes, patients in the patient-randomised control group (who received no direct patient-level interventions, but may have benefited from organisational effects at the cluster level) had better outcomes (coefficient = -2.99 95% CI -7.56 to 1.58, p = 0.186) than those in the cluster-randomised control who received neither patient- nor organisational-level intervention (coefficient = -4.64; 95% CI -7.93 to -1.35, p = 0.008). The intra-class correlation co-efficient (ICC) for our primary outcome was 0.06 (95% CI = 0.00 to 0.32). This suggests that the effect of the intervention was partly mediated through organisational effects. Therefore, we had to conclude that a patient-randomised trial of collaborative care could be vulnerable to contamination and open to type II error - underestimating the true effect size of the intervention through potential intervention 'leakage'. As a consequence, a cluster-randomised controlled trial design is the safest in order to minimise this potential source of bias and provide a truer estimate of the collaborative care intervention effect size.

### Recruitment of GP practices

We will recruit 48 GP practices in three different recruitment sites: Manchester, London and Bristol, each site responsible for the recruitment of 16 practices. All GP practices in the local Primary Care Trust (PCT) will be eligible for inclusion.

### Randomisation of GP Practices

We will allocate practices by minimisation, with a random element, on centre, deprivation and practice size. We will use the Index of Multiple Deprivation 2007 [21] to assess the level of deprivation in each practice.

## Patient Recruitment

### Sample Size

We have powered this trial to detect an effect size of 0.4. Our effect size is towards the conservative end of our platform trial confidence interval [19] SMD: 0.63; 95% CI 0.18 to 1.07, an effect size achieved by Pilling et al [22] SMD 0.42; 95% CI -0.05 to +0.89, and in line with the findings of recent reviews [23] and our group's recent meta regression [14]. An effect size of 0.4 is also regarded as the most reasonable for determining differences between interventions that are clinically meaningful [24]. Such an effect size at 90% power (alpha 0.05) would require 132 patients per group in a two armed patient randomised trial. For the proposed cluster trial, with 14 patients per cluster and the ICC found in our trial platform of 0.06, the design effect would be 1.65. The cluster trial sample size is, therefore, 440. In order to follow up 440 patients, we will recruit and randomise 550 patients to anticipate a loss to follow up of 20%.

### Patient inclusion criteria

We will include patients meeting the diagnostic criteria for depression who are aged 18 years and above and who are not currently receiving treatment for depression from specialist mental health services. We will establish the diagnosis of depression by the use of the Clinical Interview Schedule (CIS-R) [25] undertaken by a research worker. We will include both patients newly identified as depressed, with or without one or more previous depressive episodes, and those with an existing diagnosis of depression which is not responding to primary care management. We will also include patients who are suffering from peri- or post-natal depression, with either co-morbid physical illness or co-morbid non-psychotic functional disorders, such as anxiety. In line with the pragmatic nature of this trial, we will reflect usual GP care and participants will be eligible to participate whether they are in receipt of antidepressant medication or not.

### Patient Exclusion Criteria

We will exclude patients whose risk of suicide is sufficiently acute to demand immediate management by a specialist mental health crisis team. We will exclude patients with psychosis, both type I and type II bi-polar disorder, patients where the low mood is better explained by the death of someone close to them and patients whose primary presenting problem is alcohol or drug abuse. Patients who are currently receiving specialist treatment for their depression will also be excluded.

### Patient Identification and Recruitment

Cluster trials are vulnerable to selection bias through systematic differences in referral behaviour between experimental and control practices. We will minimise this potential bias by recruiting patients through searching GP records, rather than by direct GP referral. This method was also the most productive when we tested a range of recruitment methods in our pilot trial [17]. We will identify suitable patients by examining electronic case records for all patients in each general practice. The search will be limited to patients seen by their GPs in the previous four weeks who have been allocated a 'Read Code' for depression, and will be conducted by practice staff or Clinical Studies Officers blind to the random allocation of the GP surgery.

Identified patients will receive a letter from their GP surgery inviting them to take part in the study, enclosing a patient summary sheet outlining the study with a 'Permission for researcher to contact' form to allow a researcher to contact them. Recruitment which requires patients to return paper forms is known to have a very poor response rate, typically only 15% of those contacted will agree to be contacted, a threat to the study's external validity. A recent review of RCT recruitment methods [26] showed that the only likely method of improving recruitment was through telephone reminders by clinicians to non-responders. Consequently, in order to make our sample more representative of a primary care depressed population, we will attempt to enhance our response rate through telephone follow up by practice staff or Clinical Studies Officers.

### Screening and Baseline

At the screening appointment the researcher will confirm the diagnosis of depression using the CIS-R [25]. The CIS-R is a computerised interview schedule that establishes the nature and severity of neurotic symptoms and identifies a categorical diagnosis of mild, moderate or severe depression. If the patient is depressed and meets all other inclusion criteria we will collect baseline primary and secondary outcome measures.

### Allocation

Once the baseline assessments are complete, the participant's details will be entered into our automated allocation service via telephone or the internet. Each participant will be assigned an ID number and if their practice is one assigned to collaborative care then the participant's details will be automatically sent to the relevant case manager to alert them to contact this person. All new participants' details are also sent to the trial coordinator and their GP will be informed of their involvement in the study.

## Intervention - Case Management

The experimental intervention will follow the criteria identified by Gunn et al [9] for effective quality improvement strategies, which we have developed into a collaborative care protocol, tailored to UK systems and incorporating the preferences of patients, specialists and GPs from our trial platform [17,20].

Participants will receive a structured management plan including education about depression, medication management, behavioural activation and relapse prevention. The case manager will reinforce the information given to participants by their GP and by helping participants and GPs problem solve any difficulties with medication concordance, enabling participants to make good use of their medicines. Behavioural Activation (BA) is an effective cognitive-behavioural treatment of depression [27] that focuses upon reducing avoidance and increasing activity. We found BA to be acceptable in our pilot trial [20]. Relapse prevention will involve the development of individualised recovery plans which will help participants identify signature alert symptoms to prompt them to consider reinstating both their pharmacological and psychological depression management strategies.

Scheduled patient follow-ups will be organised via six to twelve scheduled telephone and face-to-face contacts by case managers with participants over a period of fourteen weeks. After an initial face-to-face contact most other contacts will be by telephone, although the option for further face-to-face contacts will be available for participants who are having difficulty settling to telephone contact. Negotiation of contact frequency will take into account participant preference, response to treatment, the requirements of the psychosocial support programme and the amount of GP-patient contact. However, in general, contacts will be weekly for the first five weeks of contact, followed by fortnightly thereafter. The initial contact session will be 30-40 minutes with subsequent sessions 15-20 minutes each.

### Case manager training and supervision

Case Managers will be either graduate psychologists or health care qualified professionals educated through existing mental health education programmes and trained specifically to deliver the collaborative care protocol. Case managers will adhere to a clinical protocol and will be supported by specialist mental health professionals who will provide weekly supervision of cases together with advice and support. Supervision for each individual participant will be no less than four weekly and will be facilitated through a bespoke computerised patient management system (PC-MIS) [28] which automatically alerts supervisors of the need to discuss all new patients, each participant at four weekly review and those participants who are not responding to treatment.

## Control intervention - Usual GP Care

Participants allocated to the control condition will receive usual care by their general practitioner. In line with the overall pragmatic approach of the trial, we will replicate 'normal GP practice' by making no specific patient-level recommendation or requirement to alter usual care by participating in the trial. GPs will treat and refer participants as would be their normal practice and participants, irrespective of their randomisation, are able to choose whether or not to take anti-depressants or ask for referral for psychological therapy. We will record every aspect of participant's usual care.

## Outcome Parameters

### Primary outcome measure

Our primary outcome will be depression severity and symptomatology as measured by the Patient Health Questionnaire-9 (PHQ9) [29] at four months. The PHQ9 is a nine-item questionnaire, which records the core symptoms of depression.

### Secondary outcome measures

We will measure quality of life using the SF36 [30], worry and anxiety by the GAD7 [31], health care utilisation using a bespoke designed patient service utilisation questionnaire, health state utilities with the EQ5D [32] and patient satisfaction with the CSQ8 [33,34]. All measures will be taken at baseline, four and twelve months follow up (see table 1), except the CSQ-8 which will only be taken at four months. In all cases, steps will be taken to ensure that the researcher who is assessing depressive symptomatology is blind to the participant's treatment arm of the trial.

**Table 1:** Outcomes and Instrument

**Outcome Parameter**	**Instrument**
**Primary Outcome**	
Depression	PHQ-9

**Secondary Outcome**	
Quality of Life	SF36
Worry and Anxiety	GAD7
Health Care Utilisation	Patient Service Utilisation questionnaire
Health State Utilities	EQ5D
Satisfaction with Care	CSQ-8

### Process Data

Process data will be collected within the trial. The extensive quantitative and qualitative work in our pilot trial has indicated that the planned intervention is effective and acceptable, and our process evaluation in this main trial will focus on (i) mechanisms of change and differential response in patient subgroups, and (ii) the process of implementation of the intervention [35]. Investigation of mechanisms of change and differential response in participant subgroups will include quantitative measurement of key participant's baseline characteristics (e.g. severity of depression; duration of depression; patient preferences; attitudes towards treatment), treatment process measures (e.g. therapeutic alliance; concordance with treatment; behavioural activity levels), contextual practice variables (e.g. anti-depressant prescription rates; availability of counselling and other mental health services) and will follow conventional procedures for analysis [36]. Investigation of the process of implementation will utilise routinely collected data from case records, session audio tapes and supervision records. The analysis will examine the implementation of collaborative care, treatment fidelity, differences between sites and different case managers and predictors of outcome.

## Analysis

### Statistical analysis of clinical data

We will analyse our primary outcome of severity of depression on the PHQ-9 at four months using between groups analysis of covariance on individual baseline depression score. Analysis will take clustering into account by use of robust standard errors in Stata. All other clinical outcome variables will be analysed as secondary variables in the same way, using least squares or ordered logistic regression as appropriate. We will analyse outcomes by site variables to detect any effects on treatment outcomes of a 'therapist effect' - differences between case managers - and practice level variables. We will investigate the effects of any missing data using imputation by best subset regression and apply CONSORT standards for cluster randomised trials [37] in data reporting.

The incremental cost per QALY of the intervention compared to the control will be calculated from NHS and Personal Social Services (PSS) perspectives following NICE evaluation guidance [38] and a wider societal perspective. The units of resource for the intervention will be collected directly and the use of other health care services by both groups will be collected through a patient questionnaire. Data on social care, other welfare services and employment details will be collected from the Patient Service Utilisation questionnaire. Intervention costs will be based on delivery costs within the trial and include supervision and appropriate capital and overhead amounts. Other unit costs will be based on long run opportunity costs and drawn from national sources. Full sensitivity analyses will be conducted with bootstrapping to provide confidence intervals around cost and effect estimates and to produce cost acceptability curves.

Qualitative data will be subject to content analysis within a qualitative methodological framework using QSR NVivo software and analysed according to the conceptual matrix of Miles and Huberman [39].

### Frequency of analyses

We will analyse data at four and twelve months follow up. The DMEC will undertake an interim data analysis to detect any reason for halting the trial.

## Ethical Issues

We will conduct the trial is such a way as to protect the human rights and dignity of the participants as reflected in Helsinki Declaration [40]. Participants will not receive any financial inducement to participate. The study has received Multi-Centre Research Ethics Committee approval from the South West Research Ethics Committee in the UK. Local Research Ethics Committee and NHS Research and Development approvals have also been given for each recruitment site. To conform to data protection and freedom of information Acts, all data will be stored securely and anonymised wherever possible. No published material will contain patient identifiable information.

## Obtaining informed consent from participants

Informed consent will be determined by a two phase consent process. Participants will receive a study information sheet in the post and a form seeking their permission to be contacted by a member of the research team, not at this stage to give consent to trial participation. Full informed consent will only be obtained through an interview by a researcher where the information sheet is fully explained and where the opportunity to ask questions is given. The opportunity to withdraw from the trial will be fully explained. Researchers seeking consent will be fully trained and supervised by the CI and site leads. Communication and recording systems will be set up to enable the trial team to monitor and act on participants' wishes to withdraw from the trial.

## Risks and anticipated benefits for trial participants and society

All participants will receive usual GP care, and therefore no treatment will be withheld to participants in this trial. This trial may in fact benefit individual participants, since collaborative care is not routinely available and has been shown to be effective in our trial platform. By participating in this trial, participants will also receive a more intensive level of monitoring than that normally received in primary care.

## Informing potential participants of possible benefits and known risks

The patient information leaflets will provide potential participants with information about the possible benefits and any known risks of taking part in the trial. Participants will be given the opportunity to discuss this issue with either their GP or trial coordinator prior to consenting to participate. The trial coordinator will inform the participants if new information comes to light that may affect the participants' willingness to participate in the trial.

### Suicide

Inherent in the nature of the condition under scrutiny (depression) is the risk of suicide and deliberate self-harm. All participants will be subject to usual GP care, and the primary care physician will be responsible for the day to day management of depression - and will ultimately be responsible for all patient-level treatment/management decisions - including prescribing, referral and assessment of risk. The pragmatic nature of this trial means that we will not seek to influence this arrangement. However, we will follow good clinical practice in monitoring for suicide risk during all researcher encounters with trial participants. Where any risk to participants due to expressed thoughts of self-harm is encountered, case managers will apply the procedures taught in the STORM training [41], and all sites will follow a local suicide protocol. Systems will be put into place to ensure that the CI, trial coordinator and researchers will be informed should there be any risks to the participants' safety.

### Trial Steering Committee (TSC) and Data Monitoring and Ethics Committee (DMEC)

A Trial Steering Committee has been set up, in addition to a data monitoring and ethics committee (DMEC). These committees will meet at least annually. The DMEC will undertake an interim data analysis to detect any reason for halting the trial.

### Forecast execution dates

The preparatory period started in September 2008, and will continue for 9 months. Recruitment will begin in June 2009 for a period of 18 months. Follow up will last 15 months, at four (T1) and 12 months (T2) after inclusion (with an additional three months of back up built in). Data analysis and reporting will take another 6 months. The entire study period will last for 48 months.

## Discussion

The current trial is designed to implement evidence based treatments within the UK primary care setting. The multiple components of collaborative care for depression have been shown to improve outcomes for patients [11-13,42]. However despite a number of collaborative care models being currently trialled in Europe [43-45] the vast majority of the evidence emerges from the US, demanding the need to test a model that is specific to the UK context. Collaborative care is a complex intervention, the development of which ideally requires a phased approach [15,16,46]. Our trial utilises this phased approach, and follows on from a previous Phase II trial which demonstrated that the collaborative care intervention can be both effective and acceptable to patients [18,20]. We have designed this Phase III cluster trial to deal with contamination issues which we explored and identified in our Phase II trial: cluster-randomised trials are recommended for situations where systems level interventions such as collaborative care are to be tested [47] since patient-randomised trials may be vulnerable to contamination from the intervention to control patients.

The outcome of this trial will have implications for the NHS, in terms of helping to improve the organisation of its care for depressed patients in primary care. We expect the results to assist primary care healthcare providers to choose how to deliver an effective model of enhanced depression service.

## Competing interests

The authors declare that they have no competing interests.

## Authors' contributions

DAR, RA, MB, JMB, PB, JC, CC-G, LG, SG, CG, DK, GL, KL, CM and SP conceived and designed the study and obtained funding. DR, AH-M and RH drafted the manuscript and all other authors contributed to editing of the final manuscript.

## Pre-publication history

The pre-publication history for this paper can be accessed here:


